# Diversity of key players in the microbial ecosystems of the human body

**DOI:** 10.1038/srep15920

**Published:** 2015-10-30

**Authors:** Ferenc Jordán, Mario Lauria, Marco Scotti, Thanh-Phuong Nguyen, Paurush Praveen, Melissa Morine, Corrado Priami

**Affiliations:** 1 The Microsoft Research - University of Trento Centre for Computational and Systems Biology, Piazza Manifattura 1, Rovereto, TN, 38068, Italy; 2 MTA Centre for Ecological Research, Karolina út 29, 1113, Budapest, Hungary; 3 GEOMAR Helmholtz Centre for Ocean Research Kiel, Duesternbrooker Weg 20, 24105 Kiel, Germany; 4 Life Sciences Research Unit, University of Luxembourg, 162 A, avenue de la Faïencerie, L-1511 Luxembourg; 5 Department of Mathematics, University of Trento, Via Sommarive 14, Povo, TN, 38123, Italy

## Abstract

Coexisting bacteria form various microbial communities in human body parts. In these ecosystems they interact in various ways and the properties of the interaction network can be related to the stability and functional diversity of the local bacterial community. In this study, we analyze the interaction network among bacterial OTUs in 11 locations of the human body. These belong to two major groups. One is the digestive system and the other is the female genital tract. In each local ecosystem we determine the key species, both the ones being in key positions in the interaction network and the ones that dominate by frequency. Beyond identifying the key players and discussing their biological relevance, we also quantify and compare the properties of the 11 networks. The interaction networks of the female genital system and the digestive system show totally different architecture. Both the topological properties and the identity of the key groups differ. Key groups represent four phyla of prokaryotes. Some groups appear in key positions in several locations, while others are assigned only to a single body part. The key groups of the digestive and the genital tracts are totally different.

The human body contains a huge variety of microorganisms. They are mostly harmless and sometimes also essential symbionts, being important for normal development and health^1^. There is an increasing knowledge about the kinds of organisms, their abundance and taxonomical distribution in various parts of the human body. However, we still have to understand much better how they function as a system: how they interact with each other, which of them play key functional roles, how sensitive their community (a set of interacting organisms competing for resources and cooperating towards a common goal) is to the loss of any of them. This information is essential for placing individual bacterial species into a system context. It seems to be increasingly recognized that only one or a few species play key roles in a large and complex networks of interactions^2^,^3^. For example, the roughly 700 species living in the oral cavity form biofilms as multispecies communities with a rich interaction network of competition and cooperation^4^. Knowledge is available about the interaction types (*e.g.*, metabolic or physical) in such ecosystems^5^,^6^. In some cases, the mechanisms of interactions are not completely clear but their effects are described at the phenomenological level (*e.g.*, *Clostridium* effects modulated by *Escherichia coli* or *Bifidobacterium*^7^).

There is an emerging interest in various ecological aspects of these microbial communities, like the functional redundancy among bacterial species^8^ or the diversity of core species sets dominating ecosystem functioning^9^,^10^. Key organisms need to be identified and placed in a community framework to understand how their behavior depends on the rest of the network and how the functioning of the whole network depends on them. Recent results suggest that the high genetic diversity of the microbiota makes the host less sensitive to a number of diseases, ranging from inflammation to metabolic disorders^11^. This diversity is variable among individuals^12^ and we have to understand also the roles of these organisms and the relationships among them (*e.g.*, their functional diversity^13^).

Interactive systems can be modelled by networks and network analysis is one of the key methodological toolkits in systems biology^14^,^15^. It has recently been used for characterizing some system-level properties of the gut microbiota. Borenstein and Feldman^16^ estimated the strength of metabolic interactions by network analysis. Turnbaugh and colleagues^17^ revealed that obesity-related genes tend to be peripheral in bacterial gene networks and they serve probably like an interface between the bacteria and the host organism. They have also found that obesity genes are less modular in the network. These findings are more about the intracellular basis of gut ecology, but similar methods can be used to study the inter-specific relationships between species. Several network analytical techniques have been used (*e.g.*, betweenness centrality^18^), and different types of networks have been constructed (*e.g.*, signed graphs^19^), but there are still possibilities to check the usefulness of several alternative network analytical methods.

We have selected 8 locations around the digestive system and 3 in the female genital organ, where data were available. We have taken a systems approach and performed network analysis in order to compare both system-level and local properties of the 11 studied microbial communities. We have identified community structure and the key organisms of the studied networks. We support earlier suggestions that ecological theory can provide a useful perspective and toolkit for better understanding microbial ecosystems^20^.

## Methods

### Data collection and network construction

The OTU census data based on 16S variable region 3–5 (V35) were downloaded from the Human Microbiome Project (http://hmpdacc.org) database providing information about the bacterial composition of human samples^21^,^22^,^23^.

In network analysis it is always very useful to take a comparative view and analyze several similarly described networks in parallel, for example, along some gradient^24^. Comparative studies on different microbial ecosystems help to understand variability and diversity, for example, across the human body^25^. Comparing several body parts can provide information that is unavailable if one looks only at a single network. We were interested in 11 locations (“body parts”) representing the microbiologically diverse digestive system and the female genital system dominated by *Lactobacillus*. These included the buccal mucosa, the hard palate, the palatine tonsils, the saliva, the tongue dorsum and the throat (proximal digestive), the stool (distal digestive), the anterior nares (spatially close to the digestive system) as well as the posterior fornix, the vaginal introitus and the mid-vagina (female genital).

First, we have filtered the data: we included only OTUs appearing in at least 20% of the samples for each particular body part, in order to increase computational efficiency and to focus on the dominant organisms. There is some risk here to omit rare but sporadically important organisms (heterogeneously in time or space), however also our analysis shows several minor groups apart from the most dominant and characteristic organisms. OTUs represent phylogenetically diverse groups: based on sequence similarity, particular OTUs can be at different levels on the phylogenetic tree, the sole criterion is their similarity. Yet, most OTUs can be relatively easily aggregated into larger groups representing well-known types of organisms (*e.g.*, genera, families).

We constructed interaction networks by the sparCC methodology^26^. SparCC infers correlations from abundance data, based on the estimations of linear Pearson correlations between log-transformed ratios of components. Only highly significant interactions (p < 0.05) have been considered. Resulting from sparCC, the edges in the interaction network are unweighted (binary) and undirected (symmetric) correlation values. This approach has several limits: correlative relationships may indicate only indirect effects, and, in general, a range of different interaction types (including metabolic linkage, physical effects) cannot be separated. Yet, the promising alternatives^27^,^28^, focusing on the dynamical coupling of abundance and functional importance, are still not easy to use on large databases (lack of time-series data, poor information on dynamical parameters). Similar techniques, quantifying key species based on dynamical ecosystem simulations and sensitivity analysis, are in infancy also in macroecology (*e.g.*^29^).

### Network analysis—global properties

The simplest properties of networks are the number of nodes (*N*) and the number of edges or links (*L*). In undirected networks, the maximum number of links (L_max_) equals


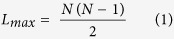


(not considering self-loops) and the density (*C*) of the network is defined as:


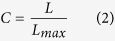


It is also of interest to see whether the neighbors of a node are neighbors of each other. Transitivity (*i.e.*, clustering coefficient) of node *i* is defined as the density of the network composed of the neighbors of node *i*. The transitivity of the network is averaged over the transitivity value of each node in the network.

The shortest path length between nodes *i* and *j* (*pl*_*(min)ij*_) is defined as the *d* distance of the two particular nodes, and the longest distance among all of the *d*_*ij*_ values is called the diameter of the network. The average of all *pl*_*(min)ij*_ minimal path length (distance) values is the average path length (*APL*) of the network. Global network statistics are presented in Table 1.

### Network analysis—local properties

First, the positional importance of network nodes is characterized and quantified by their degree (*D*) that equals the number of neighbors (*i.e.*, directly linked partners). Among many other indices, this is the most local information about the position of a network node. Thus, an OTU is considered more central if its degree is higher.

Since degree is a local network property, we decided to study also an alternative topological index providing information about the non-local, indirect neighbourhood of nodes. Betweenness centrality (*BC*; non-normalized Freeman node betweenness in the undirected graph) is used for quantifying positional importance and identifying key organisms, based on their role in transmitting indirect effects. *BC* measures how frequently species *i* lies on all shortest paths of interaction between all other species pairs. A species with high betweenness centrality is important because it mediates many indirect interactions between species. The standardized index for node *i* (*BC*_*i*_) is:


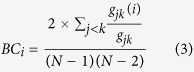


where *i *≠ *j* and *k*. *g*_*jk*_ is the number of equally shortest paths between nodes *j* and *k*, and *g*_*jk*_*(i)* is the number of these shortest paths to which node *i* is incident (*g*_*jk*_ may equal one). The denominator is twice the number of pairs of nodes without node *i*. If *BC*_*i*_ is large for the group *i*, it means that deleting this group will cause many rapidly spreading effects in the interaction network.

### Statistical analysis

We used hypergeometric test to determine whether a particular OTU type is randomly represented in the top 20% of the centrality rank for all OTUs. This was determined also at other cutoff levels (5%, 10%). However we study the statistical co-occurrence among individual OTUs as the basic category in our research, we consider OTU types as groups of organisms of biological and clinical relevance (species, genera, families). The over-representation of OTU types is suggested to indicate functional importance: over-represented OTUs are expected to appear less frequently in the particular studied fraction (*e.g.*, 20%) of the centrality rank by chance. Thus, their disproportional and statistically significantly frequent appearance is suggested to indicate some biological functioning. OTU types that are not over-represented can still be clearly numerous in the top of the centrality rank but their importance is not surprising (considering their abundance). In ecological terms, these are not keystone species but dominant species^30^.

## Results

**Figure 1:**
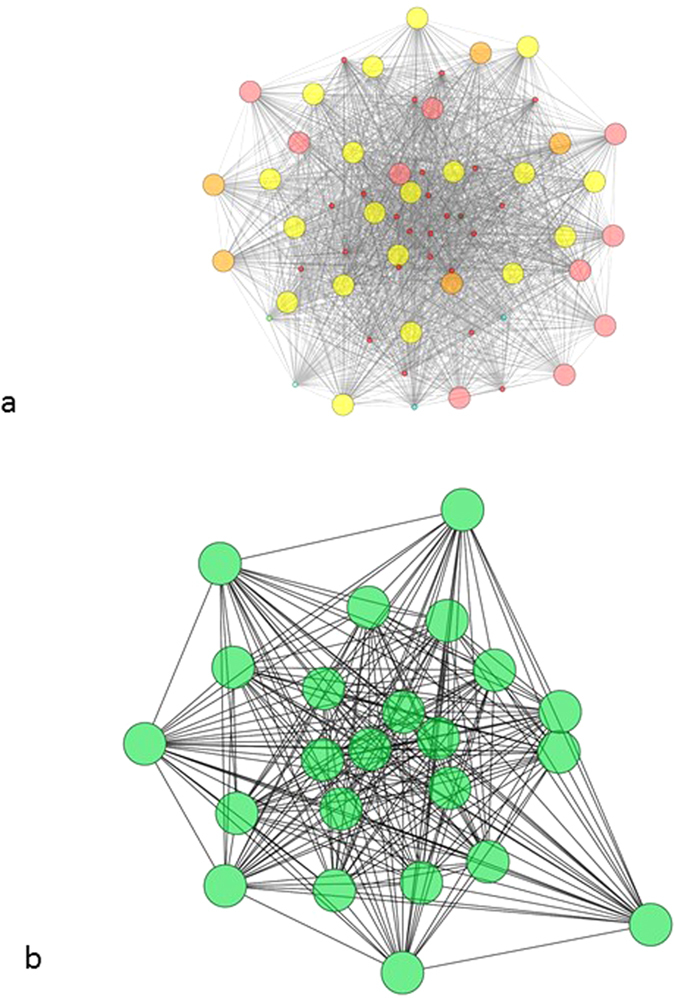
Sub-networks showing the relationships among the top 5% of the degree-based centrality rank of OTUs in the stool (a) and in the mid-vagina (b) ecosystems. The stool network includes 63 OTUs and 1680 links, while 23 OTUs and 253 interactions constitute the mid-vagina network. Large nodes stand for over-represented groups of OTUs (hypergeometric test, p < 0.05). In (**a**), we have 24 nodes for *Bacteroides* (red), 19 for *Faecalibacterium* (yellow), 10 for *Subdoligranulum* (pink), 5 for *Oscillosporaceae* (orange), 2 for *Ruminococcaceae* (light blue), and 1-1 for *Lachnospiraceae* (brown), *Blautia* (light green) and *Clostridiales* (azure). In (**b**), we see only green nodes (*Lactobacillus*, 23 nodes). To visualize the networks we adopted the edge-weighted spring-embedded layout as implemented by^63^,^64^,^65^,^66^,^67^; we used edge betweenness to weight the edges.

In Fig. 1, a simplified representation of the stool and the mid-vagina networks are shown. In the former, there is a certain diversity of key players: the structure of the network is dominated mostly by OTUs belonging to *Bacteroides*, but other OTU types are also represented in the top of the centrality rank list (*e.g., Faecalibacterium* and *Subdoligranulum*) and many of them are present in the network but not in the central positions (*i.e., Coprococcus*, see also Table 2). This is quite a similar pattern also for other local communities in the digestive tract (see Supplementary Material A). The female genital ecosystem is consistently characterized by a different pattern, the total dominance of a single OTU group, *Lactobacillus*. The central part of the interaction network is always exclusively composed of OTUs belonging to this genus, however, other OTUs also appear in more peripherial network positions (e.g., *Gardnerella* in posterior fornix, *Prevotella* in vaginal introitus and *Pseudomonas* in mid-vagina, see Supplementary Material A).

**Figure 2:**
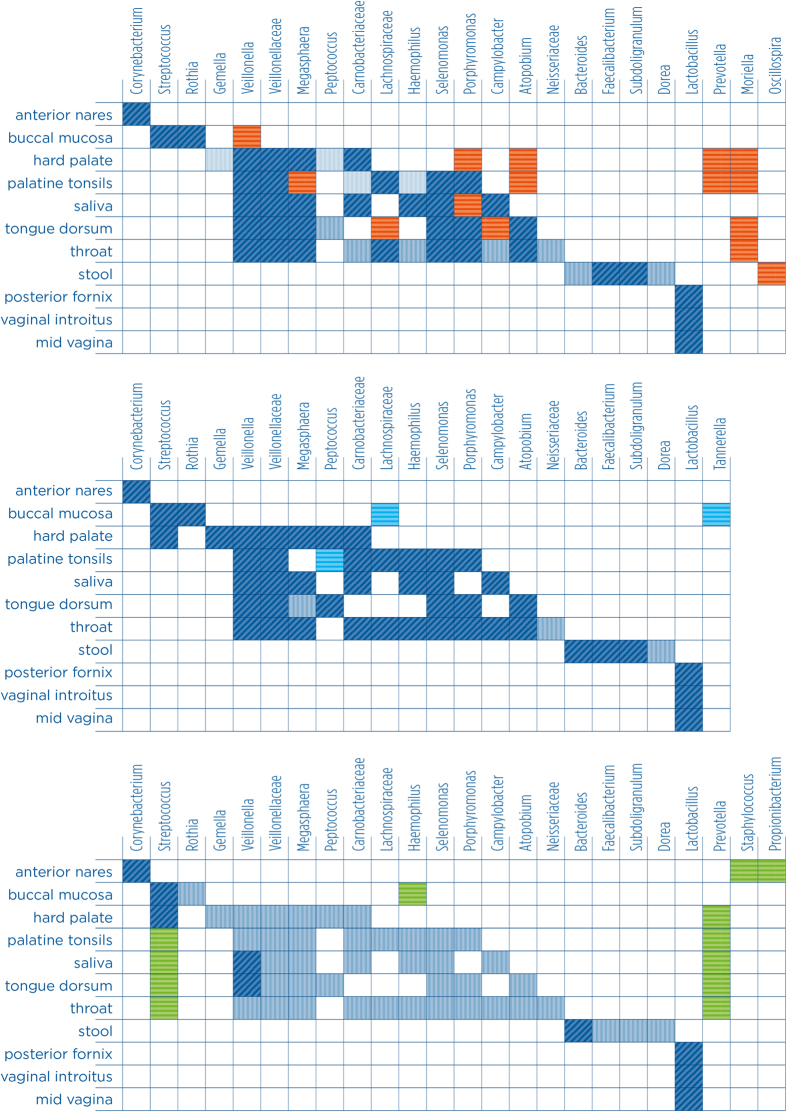
Significantly central groups of OTUs in different ecosystems (groups represented in columns and locations represented in rows). (**a**) The presence of dark blue and light blue boxes means that the type of OTU (in column) is significantly (hypergeometric test, p < 0.05) over-represented in the top 20% of the degree-based centrality rank of the interaction network of the body part (in row). If we look at the top 5% or 10% of the degree centrality rank, we may have a different set of over-represented OTU groups: red groups become over-represented, light blue groups are not over-represented anymore and dark blue groups not sensitive (remain over-represented). (**b**) If we quantify positional importance by betweenness centrality instead of degree centrality, azure groups become over-represented, light blue groups are not over-represented anymore and dark blue groups not sensitive (remain over-represented). Here, dark blue and light blue boxes correspond to the “degree/top 20%” combination. (**c**) Here we show groups that are not significantly central but clearly important, simply because of being dominant (in green): these groups provide at least 10% of the total number of OTUs. Dark blue boxes show groups that are both dominant and significantly over-represented. Light blue boxes show groups that are significantly over-represented but not dominant in this sense.

Figure 2a shows the groups of OTUs that are significantly (at p < 0.05) more frequent in the top 20% of the degree-centrality rank than random in particular locations. OTUs belonging to these groups appear in central positions quite frequently; these are considered the candidate key players in their particular ecosystems.

Based on Fig. 2 it is clear that the anterior nares, the locations in the digestive system, the stool and the genitalia are inhabited by very different communities. The anterior nares is dominated by *Staphylococcus* and *Propionibacterium* by numbers, while the structural key group is *Corynebacterium*. These organisms do not appear as central groups in any other communities. The stool is dominated by *Bacteroides*, *Faecalibacterium*, *Dorea*, *Subdoligranulum* and *Oscillospira*, and these groups do not characterize any other networks as central organisms. The female genital communities are always totally dominated by *Lactobacillus*. All of the other OTU groups are overrepresented in the central positions of networks of communities along the digestive tract.

For the sake of comparison, the interaction network of the stool ecosystem was constructed also by calculating the maximal information coefficient (MIC^31^). Our analysis is clearly based on the sparCC methodology, we only wanted to demonstrate the difference between the two approaches. We have chosen stool because the colon ecosystem is well studied and seems to be probably mostly connected to disease and health. Supplementary Material B shows these results. In case of the stool ecosystem, there is a large overlap between the key groups identified by MIC and sparCC.

The functional (biological) relevance of OTUs is sometimes in question and often unclear^32^. Their aggregation into larger taxonomic categories is always informative but problematic. The best approach seems to be performing analyses at several levels of resolution (*e.g.*^33^). In order to analyze the effects of data resolution, we have constructed an aggregated interaction network for the stool community (with *N* = 35 nodes). Supplementary Material C shows the properties of this network.

We have also checked the significantly central groups in the top 5% and top 10% of the degree-based ranking (Fig. 2a). We have used betweenness centrality as an alternative centrality measure. This allows considering the role of OTUs in mediating the spread of indirect effects in networks. Figure 2b presents the significantly over-represented groups in all studied ecosystems, based on betweenness. It also shows the differences between degree-based and betweenness-based results.

Certain groups are clearly important in particular locations, even if their centrality in the network does not differ significantly from random. These are overly abundant groups, so their frequent central position is not surprising. In order to consider these organisms, we show which groups provide at least 10% of the total number of OTUs in the top 20% of the centrality ranking. We carried out this analysis for all 11 networks (Fig. 2c).

## Discussion

Based on Fig. 2, the bacterial groups mostly belong to Firmicutes (13 groups). They provide the vast majority of bacteria in the female genital tract (*Lactobacillus*). Proteobacteria (*Haemophilus*, *Campylobacter*, and Neisseriaceae) and Actinobacteria (*Corynebacterium*, *Rothia*, and *Atopobium*) are key groups only in the proximal part of the digestive system (oral cavity and throat). Bacteroidetes (*Bacteroides*, *Porphyromonas*) play a key role in the distal digestive tract (stool) but also in the oral cavity. No clear relationship is seen between key players and enterotypes^34^. The unclear relationship between phylogeny and ecology is supported by the finding that metabolic pathways can be surprisingly similar even if taxonomic compositions markedly differ^35^.

Table 1 shows the numerical values of global network properties for each of the 11 ecosystems. The female genital system differs from the communities in the digestive tract in all respects. It is composed of a smaller number of OTUs (nodes) and interactions (edges) but its density and transitivity are higher. Even if the average path length is shorter in the genital networks, they have longer diameter, which is due to a few particular relationships. These parameters would suggest a structurally homogeneous network, yet, it is totally dominated by OTUs belonging to *Lactobacillus* (there is finer-scale variability for the following groups, see^36^). Although the global topological properties for the three female genital ecosystems and the other ecosystems markedly differ, these analyses are more like providing a general overview and not suitable for making smaller-scale, accurate and testable predicitons (*e.g.*, making a statistical difference between buccal mucosa and hard palate). Only the extreme values of global topology seem to be predictable, similarly to macroecology, where small differences in food web connectance are not easy to interpret.

The Lachnospiraceae group is widespread and appears to be highly central in several different locations in the proximal digestive tract: mostly in palatine tonsils and throat, but also in tongue dorsum (Fig. 2a) and buccal mucosa (Fig. 2b). This supports earlier findings on their widespread nature^37^.

*Faecalibacterium* is a reportedly widespread commensalist^38^. We have found in significantly central key positions only in the stool ecosystem (although its role is clearly large there).

*Bacteroides* clearly dominate the distal segments of the digestive system. As it was suggested earlier^9^, some species belonging to this group (*e.g.*, *Bacteroides thetaiotaomicron*) act as functionally highly important, keystone species. In ecological terms, keystone species have a disproportionately large effect on the community, considering their biomass^30^. These species can be outstandingly important because of several types of mechanisms, however their biomass is not easily quantified. *Bacteroides thetaiotaomicron* seems to stabilize the ecosystem by diet switch. *Bacteroides* are abundant all over the gut^35^, yet, our results show that they are in statistically even more important positions than expected randomly (Fig. 2, Table 2).

*Prevotella* is not a key group in any of the studied ecosystems (Fig. 2) but if the top group of central nodes is smaller (*i.e*., if we only focus on top 5% or 10% in the ranking; see Fig. 2a) or betweenness centrality is used instead of degree (Fig. 2b) this group is also central almost across the whole proximal digestive system (in 5 locations). Interestingly, it never overlaps with *Bacteroides*, which is a within-body example for their separation, apart from the well-documented between-body alternation caused by different dietary habits: *Prevotella* is well known to dominate in the intestinal flora of African children (because of the carbohydrate-rich diet) but it is only poorly present in European and North-American children’s digestive system (because of the protein-rich diet; see^39^). This calls for a future improvement of our analysis by studying heterogeneous samples (for diet but also geography and age, see^40^, or delivery, see^41^).

Following the totally dominant *Bacteroides*, three of the other four key groups indicated in the stool ecosystem (*Faecalibacterium*, *Subdoligranulum* and *Oscillospira*) form a key core set of organisms involved in inflammatory bowel disease (IBD) development^42^. These three organisms together seem to be crucial for healthy state vs dysbiosis in Crohn disease patients. Their key positions may be related to this ecosystem function.

Interestingly, the two groups (*Streptococcus*, *Prevotella*) suggested to be dominant in the throat ecosystem^43^ are indicated only by betweenness centrality (Fig. 2b). This suggests the importance and indicator value of this index. The results of^35^, emphasizing the abundance of *Streptococccus* in the oral ecosystem are supported but we have also found that their outstanding structural importance depends on the particular location: *Streptococcus* is outstandingly central in the buccal mucosa and the hard palate but it is not over-represented in the palatine tonsils, in the saliva, in the tongue dorsum and in the throat ecosystems (within the digestive tract). We also support its particular dominance in the oral ecosystem: in the stool, in the anterior nares and in the female genital systems it is not dominant (Supplementary Material A).

The dominance of *Lactobacillus* in the vagina ecosystems is well known and also its ability to keep the stability and resistance of the community against colonizers^44^. Its central network position clearly supports this functional importance. We can say that, according to classical ecological terms, *Lactobacillus* is a dominant species (not a real keystone species) in a low-diversity, stable community^45^. Its role in other body parts is clearly less important (not indicated in our study anywhere else as a key group) but not negligible (see^46^).

Faust and colleagues^35^ suggested that *Porphyromonas* is a negative hub in several locations. We have not considered the sign of co-occurences, but even so we can support that these organisms are never dominant, yet, over-represented in the structure of the ecosystem in several locations (palatine tonsils, tongue dorsum, throat) but not in others (buccal mucosa, anterior nares, stool, genital systems, see Supplementary Material A). In some location, their over-representation depends on the threshold level used (hard palate, saliva, see Fig. 2).

Similarly, earlier studies^35^ suggested that *Selenomonas* is one of the organisms that can structurally act as a hub without being dominant (they mentioned the tooth plaque ecosystem). We support this finding: *Selenomonas* is over-represented in the structure of four locations (palatine tonsils, saliva, tongue dorsum, throat) but not in others (see Fig. 2 and Supplementary Material A). At the same time it is not dominant in any place. Similarly, *Atopobium* was suggested to have the same character in the tongue and we found its over-representation in the tongue dorsum and the throat, without being dominant (see Fig. 2 and Supplementary Material A).

It is important to emphasize that we pooled data from several individuals. This means that we could perform a statistically robust analysis but we could not study anything related to individual-level variability in humans^12^. We recognize this is a limitation of this study but the major advantage is the robustness of the results. Documenting variability is the basis for comparing different ecosystems and better understanding their variability also in terms of the key groups responsible for the main ecosystem functions. Different characteristic groups of co-occurring species can be replaced by each other, ensuring a certain amount of functional redundancy in these microbial ecosystems^47^. In order to more clearly see the functional roles of individual OTUs, strains and species, an outlook to metabolomics and metaproteomic studies is needed^48^.

In this manuscript, we have focused only on the microbial community. However, the real ecosystem contains not only microbe-microbe but also host-microbe interactions^49^. Host-microbe relationships are crucial for both nutrition and medical research^50^. Nevertheless, better understanding a subsystem is a useful step before scaling our interest up to the whole host-microbiota system (especially because studying also fungi and Archaea could add interesting details^51^).

Better understanding the systems behavior of the microbial community will also be helpful for planning and managing pharmaceutical applications^52^,^53^. Identifying key groups (*e.g.*, the most central ones, see Fig. 2, Supplementary Material A) and key interactions (*e.g.*, see Supplementary Material C) provides testable predictions on the organisms that are of key importance in the functioning in these microbial ecosystems. This systems view offers predictions that are complementing earlier ones based on the composition and abundance of microorganisms. The diagnostic value of the gut microbiota is especially high, considering its variability, flexibility and responsiveness^54^. Quantifying and characterizing the response of the microbiota under certain conditions, cancers and other diseases (IBD^55^) or infections^56^ provide an ecological basis for applications. Predicting invasion success^57^, describing community diversity^57^ or modelling dysbiosis^58^ are all ecological problems of the microbiota. For nutritional sciences, it is essential to better understand the role individual species play in the gut ecosystem (see the selective approach of prebiotics versus the general approach of probiotics^59^,^60^. The ecological perspective is surely promising for future applications that focus on whole-community selection (*e.g.,* treatment^61^ and management^62^).

## Additional Information

**How to cite this article**: Jordán, F. *et al.* Diversity of key players in the microbial ecosystems of the human body. *Sci. Rep.* **5**, 15920; doi: 10.1038/srep15920 (2015).

## Supplementary Material

Supplementary Materials

## Figures and Tables

**Table 1:** Global network properties of the studied microbial ecosystems: the number of nodes, the number of edges, density, diameter, average path length (APL) and transitivity.

network	nodes	edges	density	diameter	APL	transitivity
anterior nares	618	72136	0,3784	2	1,6216	0,4709
buccal mucosa	1064	160541	0,2839	2	1,7161	0,3747
hard palate	1281	236289	0,2882	2	1,7118	0,3872
palatine tonsils	1356	324713	0,3535	2	1,6465	0,4717
saliva	1560	326758	0,2687	2	1,7313	0,3826
tongue dorsum	1438	404466	0,3915	2	1,6085	0,5194
throat	1342	318287	0,3537	2	1,6463	0,4737
stool	1254	293541	0,3736	2	1,6264	0,4314
posterior fornix	462	49518	0,4650	2	1,5350	0,7040
vaginal introitus	493	59645	0,4918	3	1,5082	0,6700
mid vagina	466	62086	0,5730	3	1,4270	0,7368

**Table 2:** Groups of OTUs composing the interaction network of the stool ecosystem.

stool	total	20%	D_P	B	B_P	10%	D_P	5%	D_P
		D				D		D	
Akkermansia	1	0	0,2002	0	0,2002	0	0,0997	0	0,0502
Alistipes	72	4	0,9996	3	0,9999	1	0,9961	0	0,9781
Anaerotruncus	1	0	0,2002	0	0,2002	0	0,0997	0	0,0502
Bacteroidales	4	0	0,5912	0	0,5912	0	0,3433	0	0,1865
Bacteroides	640	157	0,0000	164	0,0000	67	0,2426	24	0,9764
Bifidobacterium	2	0	0,3604	0	0,3604	0	0,1895	0	0,0980
Blautia	20	1	0,9326	1	0,9326	1	0,6085	1	0,2658
Clostridiales	13	2	0,4995	1	0,7683	1	0,3775	1	0,1358
Clostridium	21	3	0,6321	1	0,9440	2	0,3497	0	0,6643
Coprococcus	9	0	0,8670	0	0,8670	0	0,6126	0	0,3721
Dialister	7	0	0,7915	0	0,7915	0	0,5214	0	0,3035
Faecalibacterium	94	37	0,0000	34	0,0000	25	0,0000	19	0,0000
Holdemania	1	0	0,2002	0	0,2002	0	0,0997	0	0,0502
Lachnospira	12	0	0,9323	0	0,9323	0	0,7180	0	0,4628
Lachnospiraceae	50	5	0,9554	5	0,9554	1	0,9681	1	0,7292
Odoribacter	10	0	0,8938	0	0,8938	0	0,6515	0	0,4039
Oscillospira	51	14	0,0670	12	0,2032	8	0,0593	5	0,0385
Parabacteroides	65	0	1,0000	1	1,0000	0	0,9991	0	0,9680
Porphyromonadaceae	3	0	0,4886	0	0,4886	0	0,2704	0	0,1434
Rikenellaceae	3	0	0,4886	0	0,4886	0	0,2704	0	0,1434
Roseburia	40	3	0,9735	4	0,9277	1	0,9219	0	0,8770
Ruminococcaceae	27	4	0,6550	3	0,8212	4	0,1230	2	0,1502
Ruminococcus	43	2	0,9959	2	0,9959	0	0,9899	0	0,8952
Subdoligranulum	36	18	0,0000	20	0,0000	14	0,0000	10	0,0000
Alcaligenaceae	7	0	0,7915	0	0,7915	0	0,5214	0	0,3035
Burkholderiales	1	0	0,2002	0	0,2002	0	0,0997	0	0,0502
Catabacteriaceae	1	0	0,2002	0	0,2002	0	0,0997	0	0,0502
Dorea	1	1	0,0000	0	0,2002	0	0,0997	0	0,0502
Lachnobacterium	2	0	0,3604	0	0,3604	0	0,1895	0	0,0980
Sutterella	2	0	0,3604	0	0,3604	0	0,1895	0	0,0980
Turicibacteraceae	1	0	0,2002	0	0,2002	0	0,0997	0	0,0502
Eubacterium	6	0	0,7390	0	0,7390	0	0,4681	0	0,2665
Phascolarctobacterium	6	0	0,7390	0	0,7390	0	0,4681	0	0,2665
Collinsella	1	0	0,2002	0	0,2002	0	0,0997	0	0,0502
Escherichia	1	0	0,2002	0	0,2002	0	0,0997	0	0,0502

The column “total” shows how many individual OTUs belong to a particular group. Column “D” shows the number of OTUs being part of the top 20% of the centrality rank based on degree centrality and column “D_P” shows whether it is significant according to a hypergeometric test. We also show the same results based on betweenness centrality (B, B_P) as well as the degree-based results for the top 10% and 5% of the centrality rank (significant values always shaded). This network is composed of 1254 OTUs so the top 20% means 251 OTUs. Among them, there are 14 *Oscillospora* OTUs out of the 51 total *Oscillospora* OTUs, this is not significant. In the top 5% we only have 63 OTUs and the 5 *Oscillospora* OTUs in this top 5% is already significantly over-represented compared to random. Note that *Dorea* is technically speaking significant (1 out of 1 OTU in top 20%) but this should be considered with care. Similar results for other body parts are shown in Supplementary Material A.
