# Supplementary material for: Diversity of key players in the microbial ecosystems of the human body
Source: Sci Rep. 2015 Oct 30;5:15920. doi: 10.1038/srep15920 (PMC4626846; doi:10.1038/srep15920)
Supplement: Supplementary Materials [file srep15920-s1.doc]

**Diversity of key players in the *microbial* ecosystems of the human body**

Ferenc Jordán^1,2^,*, Mario Lauria^1^, Marco Scotti^1,3^, Thanh-Phuong Nguyen^1,4^, Paurush Praveen^1^, Melissa Morine^1,5^, Corrado Priami^1,5^

^1^ The Microsoft Research - University of Trento Centre for Computational and Systems Biology, Piazza Manifattura 1, Rovereto, TN, 38068, Italy

^2^ MTA Centre for Ecological Research, Karolina út 29, 1113, Budapest, Hungary

^3^ GEOMAR Helmholtz Centre for Ocean Research Kiel, Duesternbrooker Weg 20, 24105 Kiel, Germany

^4^ Life Sciences Research Unit, University of Luxembourg, 162 A, avenue de la Faïencerie, L-1511 Luxembourg

^5^ Department of Mathematics, University of Trento, Via Sommarive 14, Povo, TN, 38123, Italy

* Corresponding author: [jordan.ferenc@gmail.com](mailto:jordan.ferenc@gmail.com)

**Supplementary Material A.** Results of Table 2 are presented here for the further body parts. Note that “NA” means sequence not assigned to any particular category.

**Supplementary Material B.** Comparative analysis of stool networks constructed using sparCC and MIC methodologies. MIC (maximal information coefficient [31]) measures dependence between every pair of OTU based on co-occurrence in the census data. Detecting significant co-occurrences among members of a population quantified as relative abundances can be notoriously difficult, as the absolute increase in one organism's abundance can result in an apparent relative decrease of all other abundances; however, the MIC approach has been shown to work well for microbial relative abundance data. A fast implementation of the MIC was used [64] to generate an all-to-all MIC matrix, from which several co-occurrence networks were derived using the threshold *t* = 1.5 for percentile MIC values (we have also checked other *t* values: 0.5, 0.75, 1, 1.25, 1.75, 2, 2.25, 2.5, and 2.75).

Based on the top 20% of nodes in the degree-based ranking of the MIC-based network, *Bacteroides* and *Faecalibacterium* are significantly overrepresented among the most central OTUs. *Subdoligranulum* and *Dorea* do not appear among the significantly central groups (unlike in the sparCC-based network). However, *Blautia*, *Clostridium* and *Eubacterium* appear in the top 20% ranking based on betweenness centrality (blue).

**Supplementary Material C.** The stool ecosystem has been analyzed also in an aggregated version. This network contains *N* = 35 components: *Akkermansia* (Akk), Alcaligenaceae (Alc), *Alistipes* (Ali), *Anaerotruncus* (Ana), Bacteroidales (BacL), *Bacteroides* (BacD), *Bifidobacterium* (Bifi), *Blautia* (Blau), Burkholderiales (Burk), Catabacteriaceae (Cata), Clostridiales (CloL), *Clostridium* (CloM), *Collinsella* (Coll), *Coprococcus* (Copr), *Dialister* (Dial), *Dorea* (Dor), *Escherichia* (Esch), *Eubacterium* (Eub), *Faecalibacterium* (Faec), *Holdemania* (Hold), *Lachnobacterium* (LachM), *Lachnospira* (LachS), Lachnospiraceae (LachC), *Odoribacter* (Odor), *Oscillospira* (Osc), *Parabacteroides* (Para), *Phascolarctobacterium* (Phas), Porphyromonadaceae (Por), Rikenellaceae (Rik), *Roseburia* (Ros), Ruminococcaceae (RumC), *Ruminococcus* (RumS), *Subdoligranulum* (Sub), *Sutterella* (Sut), Turicibacteraceae (Tur).


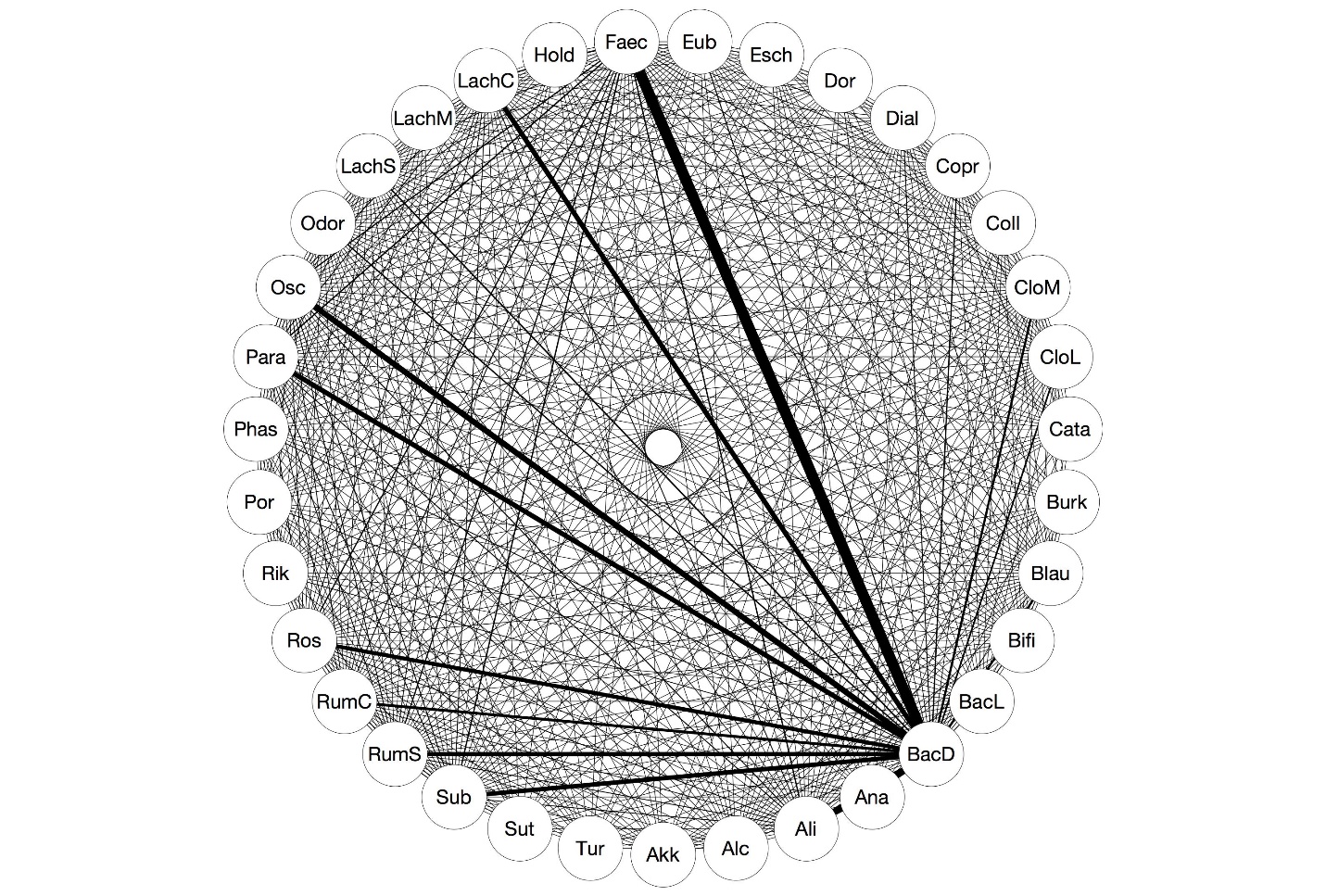


Here, OTUs have been aggregated into larger taxonomic groups, typically genera or families

(e.g. all the OTUs annotated as *Subdoligranulum* merged into one entity with the same name). These larger units provide a more general and less detailed description. It is noted that some results of studies on interaction networks depend on the level of resolution [65]. This aggregated network has been weighted by the number of significant relationships detected between variable OTUs belonging consistently to a particular pair of larger units. For example, in the original database, there were 39 associations between various *Blautia* and *Sutterella* OTUs, 3 of them being significant, thus the weight of the interaction between the aggregated groups *Blautia* (Blau) and *Sutterella* (Sut) equals 3.

Although the binary network is an almost complete graph (*i.e.*, almost all possible edges are realized), the centrality of nodes can be informative in the weighted network. We use the weighted version of the topological importance index (calculated for up to three steps of indirect effects, *WI^3^* [66]). *WI* considers indirect effects (not like *D* that focuses on direct neighbors only) and all pathways (not like *BC*, focusing only on shortest paths). This network index has been calculated using the Graph software [67].

The centrality rank of the 35 components, based on the *WI^3^* index is shown below. *Bacteroides* and then *Faecalibacterium* are of outstanding positional importance in the network. *Sutterella* is among the structurally least important groups.

| **groups** | **WI^3^** |
| --- | --- |
| BacD | 0.4056 |
| Faec | 0.1117 |
| Ali | 0.0686 |
| Osc | 0.0562 |
| Para | 0.0481 |
| LachC | 0.0475 |
| Sub | 0.0432 |
| RumS | 0.0403 |
| Ros | 0.0379 |
| RumC | 0.0249 |
| Blau | 0.0187 |
| CloM | 0.0185 |
| CloL | 0.0122 |
| LachS | 0.0111 |
| Odor | 0.0092 |
| Copr | 0.0083 |
| Eub | 0.0057 |
| Dial | 0.0045 |
| Phas | 0.0036 |
| Alc | 0.0034 |
| BacL | 0.0032 |
| Por | 0.0027 |
| Rik | 0.0026 |
| Bifi | 0.0020 |
| LachM | 0.0020 |
| Dor | 0.0013 |
| Coll | 0.0011 |
| Hold | 0.0011 |
| Ana | 0.0008 |
| Cata | 0.0008 |
| Esch | 0.0008 |
| Akk | 0.0008 |
| Burk | 0.0006 |
| Tur | 0.0006 |
| Sut | 0.0006 |

The rank of the strongest (undirected) interactions among the groups of the aggregated network. Based on the *WI* methodology, the normalized interaction strengths can be determined based on direct and indirect effects spreading along pathways of three or less steps. Indirect interactions connect all groups to all others, in this case the interaction matrix is full; we only show the strongest 25 ones. The *Bacteroides*/*Faecalibacterium* interaction is the strongest one (BacD/Faec), while the *Faecalibacterium*/*Alistipes* (Faec/Ali) interaction is the strongest one between groups other than *Bacteriodes*.

| **group 1** | **group 2** | **interaction strength** |
| --- | --- | --- |
| BacD | Faec | 0.044913 |
| BacD | Ali | 0.02758 |
| BacD | Osc | 0.022596 |
| BacD | Para | 0.019356 |
| BacD | LachC | 0.01909 |
| BacD | Sub | 0.017362 |
| BacD | RumS | 0.016187 |
| BacD | Ros | 0.015226 |
| BacD | RumC | 0.010019 |
| Faec | Ali | 0.007578 |
| BacD | Blau | 0.00754 |
| BacD | CloM | 0.007453 |
| Faec | Osc | 0.006211 |
| Faec | Para | 0.005325 |
| Faec | LachC | 0.00525 |
| BacD | CloL | 0.00505 |
| Faec | Sub | 0.004776 |
| BacD | LachS | 0.004459 |
| Faec | RumS | 0.004452 |
| Faec | Ros | 0.004188 |
| Ali | Osc | 0.003813 |
| BacD | Odor | 0.003712 |
| BacD | Copr | 0.003319 |
| Ali | Para | 0.003269 |
| Ali | LachC | 0.003223 |

Comparing this aggregated network to the original network containing OTUs as nodes, we can say that the aggregation did not have a large effect on the identity of key groups in the stool ecosystem. While the global properties of the network clearly changed, the organisms in key positions remained the same. The stool ecosystem is clearly dominated by *Bacteroides* and *Faecalibacterium*.
